# Towards a phylogenetically informed approach to solving protein–protein interactions

**DOI:** 10.1042/BST20253031

**Published:** 2025-12-03

**Authors:** Chun Shen Lim, Peter Mace, Peter C. Fineran, Paul P. Gardner

**Affiliations:** 1 Department of Biochemistry, Faculty of Biomedical Sciences, University of Otago, Dunedin, 9016, New Zealand; 2 Maurice Wilkins Centre for Molecular Biodiscovery, University of Otago, Dunedin, 9016, New Zealand; 3 Genetics Otago, University of Otago, Dunedin, 9016, New Zealand; 4 Department of Microbiology and Immunology, Faculty of Biomedical Sciences, University of Otago, Dunedin, 9016, New Zealand; 5 Bioprotection Aotearoa, University of Otago, Dunedin, 9016, New Zealand

**Keywords:** artificial intelligence, evolutionary conserved protein–protein interactions, machine learning, protein complex prediction

## Abstract

Protein–protein interactions (PPIs) are critical to all cellular activities. Despite having a large number of proteins, cells have spatial and temporal control over PPIs to avoid dysregulation in cellular pathways. Considerable research efforts have aimed to find new PPIs, curate PPIs from the literature and build searchable PPI databases. These databases have been widely used by experimental and computational scientists. Here we find that the PPIs captured by these databases are highly heterogeneous and concentrated on a small number of species. These issues hamper researchers from capturing the full landscape of reliable PPIs, affecting the accuracy of machine-learning models and the effectiveness of experimental designs. However, there are opportunities to fill gaps computationally and experimentally. We suggest developing a phylogenetically informed approach to test PPIs experimentally and computationally.

## Introduction

Protein–protein interactions (PPIs) drive key cellular processes, including replication, transcription, RNA processing and translation, and contribute to complex regulatory networks. They are also important for studying disease states, as many diseases are due to aberrant PPIs [1,2]. This has motivated various small- and large-scale experiments in probing the number and type of PPIs across different model organisms, which are captured by several publicly available databases [3].

However, in a crowded cellular environment, the number of unique, non-functional, stochastic interactions between different proteins grows quadratically with proteome size (specifically, following the binomial coefficient nC2). For example, *Escherichia coli* and *Homo sapiens* have roughly 4K and 20K proteins respectively, and there are approximately 8M and 200M potential unique one-to-one interactions respectively. Yet, only a modest fraction of all possible PPIs are biologically relevant, suggesting that many interactions are the non-functional products of a crowded cellular environment [4 12]. This means distinguishing functional from non-functional interactions is critical, particularly for complex species with a large cohort of proteins.

Proteins can form homo- or hetero-oligomeric complexes through transient or permanent interactions, which may be obligate (essential for a complex’s function) or non-obligate [13,14]. Dynamic oligomeric states, such as those in the translation initiation complex eIF3 or the spliceosome, typically involve transient interactions [15,16]. Therefore, interaction strength is not equivalent to functional importance. Unlike transient interactions, permanent interactions—such as those in steroid receptors and HIV-1 pre-integration complexes—often result in unstable monomers when not bound in the complex [17 20]. These permanent interactions are typically essential for both the structural integrity and function of the complex.

Various experimental and computational methods have been developed to detect PPIs [10,21,22]. Common techniques include *in vitro* methods (e.g., GST-pulldown) and *in cellulo* methods (e.g., proximity ligation assay [23,24] and yeast two-hybrid genetic system [25]). Some other experimental techniques combine *in cellulo* and *in vitro* methods (co-immunoprecipitation [26] and mass spectrometry-based approaches [8]). Recently, advanced machine learning approaches leveraging sequence and coevolutionary information have gained significant traction, such as those implemented in AlphaFold3 and ESM3 [27,28]. *In silico* and experimental methods have also been used in conjunction to characterise PPIs [8]. Moreover, numerous innovative techniques have been developed to improve PPI detection, e.g., novel assays based on colocalisation and protein-fragment complementation [29 32].

**Figure 1:**
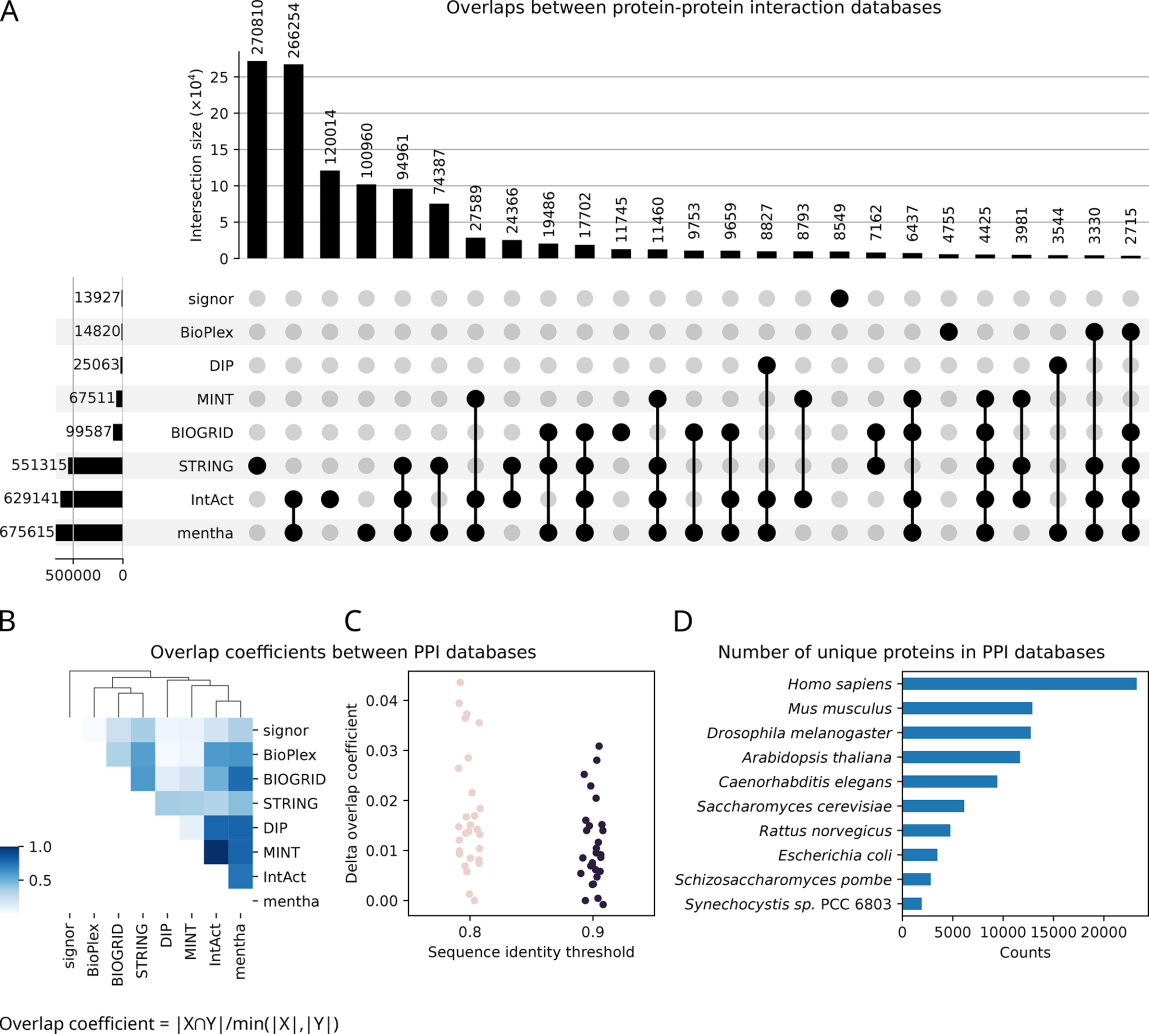
Sizes and distributions of protein–protein interaction (PPI) databases. **(A**) An UpsetR plot illustrating the overlap between PPI databases based on unique primary UniProt IDs. Databases are listed on the left of the lower panel. The black dots indicate intersections between different databases. The bars to the left show the database sizes, and the bars above indicate the overlap size (e.g., STRING holds 680K PPIs, 313K unique to STRING). Physical-only PPI releases were used whenever possible, as for BioGRID and STRING. (**B**) Overlap coefficients between PPI databases (left panel). To rule out the possibility of ID mis-assignments, homology clustering of protein sequences was performed using the amino acid sequence identity thresholds of 0.8 and 0.9. The differences between the overlap coefficients before and after clustering are negligible (right panel). (**C**) Counts of the unique proteins (UniProt IDs) for each species for PPIs that were found in two out of eight databases (the 10 most common species are shown).

The collective efforts in solving PPIs have generated a substantial amount of PPI data (Figure 1), which is available in the literature. While remarkable progress has been made in recent years, many challenges persist. The large-scale identification of PPIs can generate a high number of false positives, making it hard to distinguish functional interactions from random noise [7,32,33]. Therefore, efforts have been made to develop and curate PPI databases, including BioGRID [34], DIP [35], IntAct [35,36], mentha [37], MINT [38], STRING [39], and SIGNOR [40]. These databases provide metadata on the source publications, species studied and experimental methods, helping researchers assess the reliability and relevance of each PPI entry.

**Figure 2:**
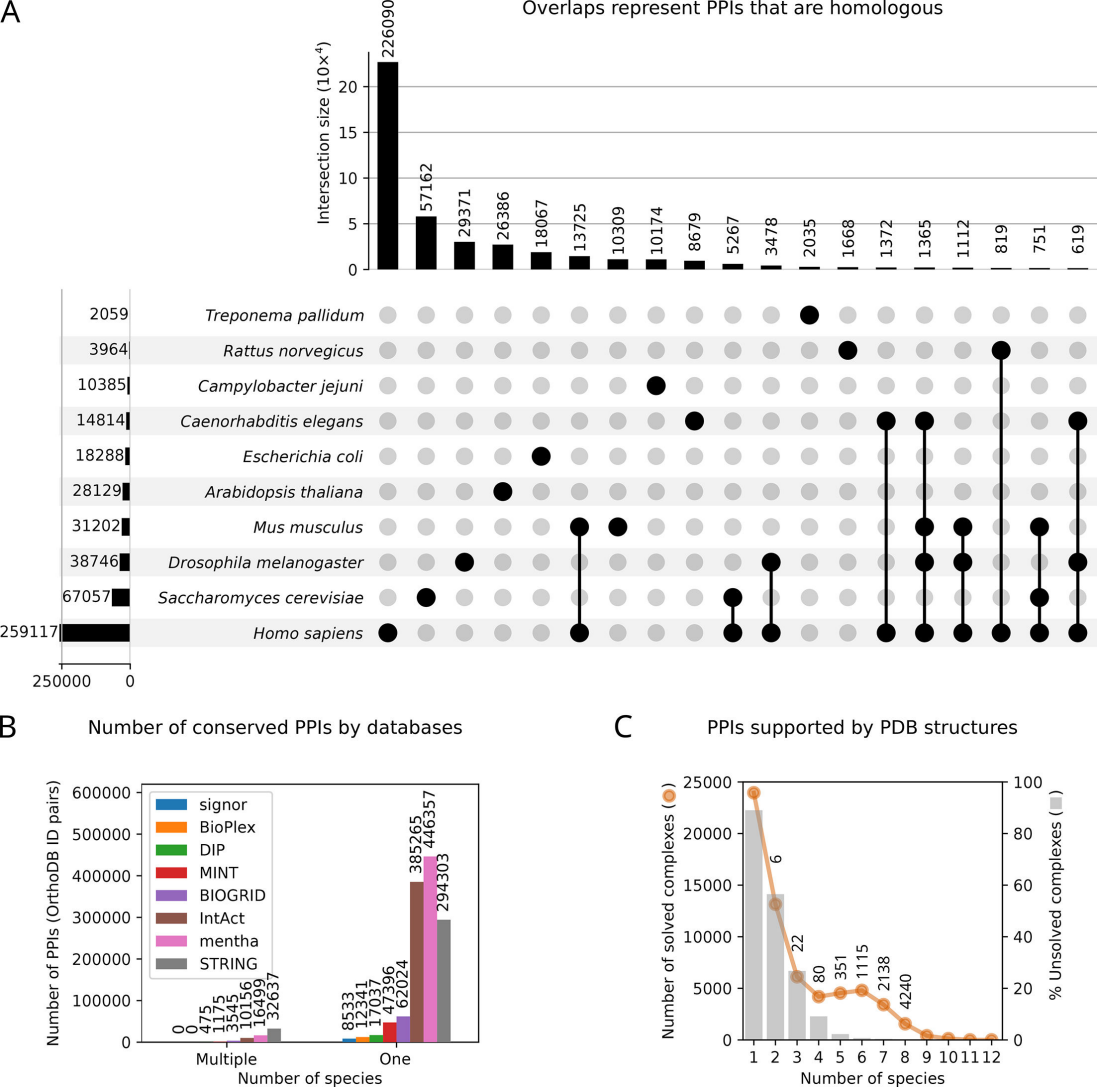
Conserved PPIs were sourced from eight PPI databases. **(A**) UpSet plot shows the conservation of PPIs, based on the overlap of homologous protein pairs (OrthoDB ID pairs) across species (NCBI taxonomy IDs). Dots with vertical edges connecting them indicate a union between sets, while the vertical bar plot above indicates the size of the union (e.g., *Homo sapiens* and *Mus musculus* share 13,643 PPIs). (**B**) The number of conserved PPIs hosted in the eight PPI databases. The proteins are grouped by orthology, where PPIs that share the same pairs of OrthoDB IDs in multiple species are defined as evolutionary conserved. (**C**) Many PPIs are not represented in the PDB structure database. Notably, structurally characterised PPIs are overrepresented among highly conserved interactions compared with less conserved ones, as indicated by the odds ratios above the points (6, 22, 80, etc). For example, for conserved PPIs in two species, the odds of finding corresponding complex structures deposited in the PDB are six times higher than that of non-conserved PPIs. Moreover, for conserved PPIs found in 6 or more species, > 97% of these have a corresponding PDB structure, whereas for 5 species there are 35 PPI structures remaining to be solved (see Figures 3 and 4). PDB, Protein Data Bank.

However, here we find that the PPIs captured by these databases are variable, sparse and focused on model organisms. Several efforts, such as HitPredict [41] and ConsensusPathDB [42], have aimed to unify PPI databases, and these meta-databases may help fill some gaps. However, these meta-databases do not address the issue that most PPIs have been identified in a limited number of species. Encouragingly, we find that protein complexes with experimentally determined structures are enriched in the highly conserved PPIs (Figure 2), suggesting an opportunity to use structural and *in silico* approaches to further explore conserved PPIs.

**Figure 3:**
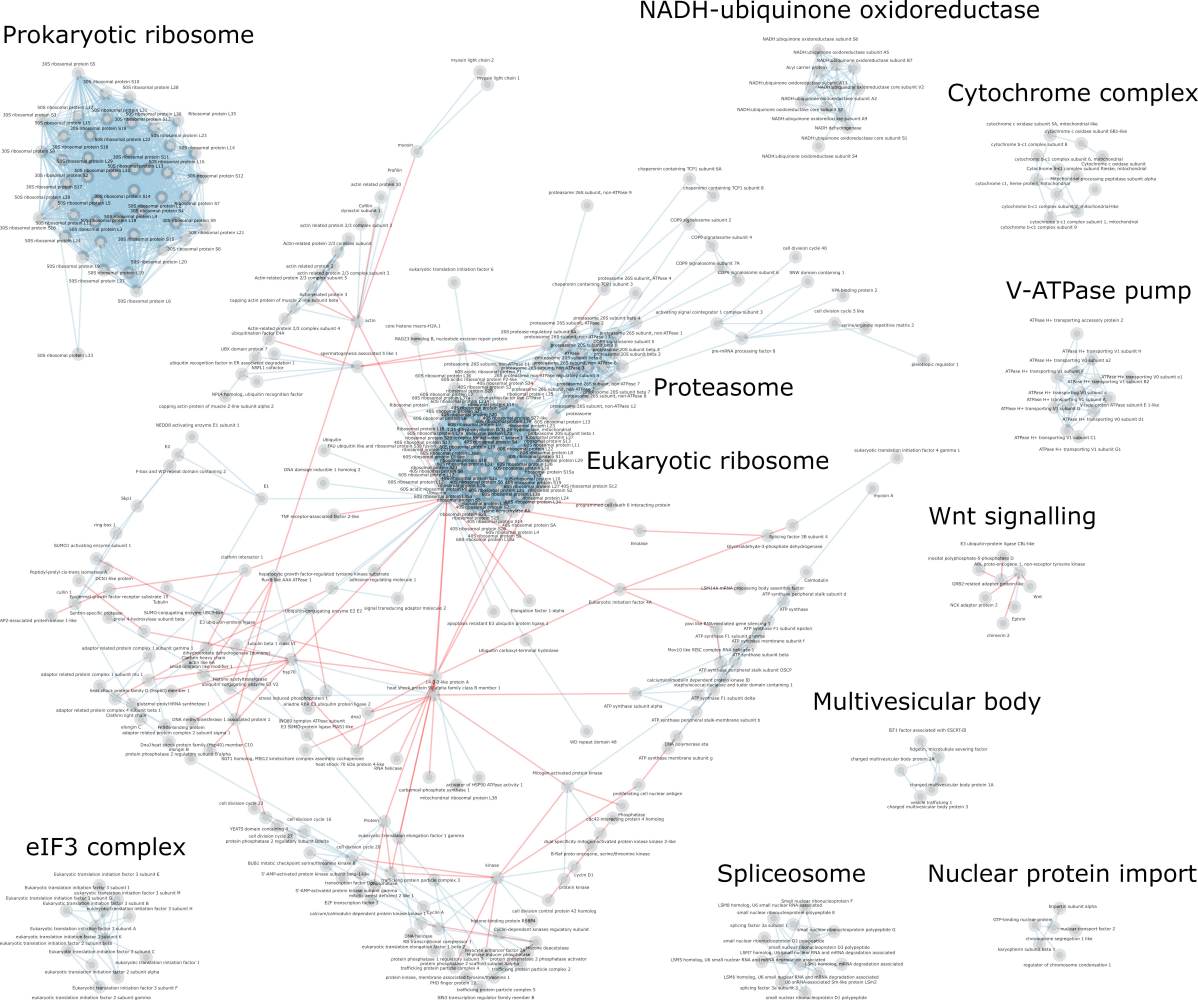
The 10 largest conserved PPI networks. The nodes represent orthologous protein groups (by OrthoDB IDs). The blue and red edge colours represent structurally solved (with PDB IDs) and unsolved PPIs, respectively. Only PPIs observed in at least five species are shown.

We propose broadening the scope of PPI analysis to include a broader range of species. A phylogenetically informed approach inspired by the successes of phylogenetically informed data acquisition through genome sequencing, such as the GEBA (Genomic Encyclopedia of Bacteria and Archaea ) and Zoonomia projects, is promising [43 47]. Prior work on estimating the statistical power of comparative genomic methods showed that the detection of smaller conserved elements improves as more genome sequences are added [48,49]. We anticipate that these seminal studies will have important implications for PPI analysis, similar to their impact on comparative genomics during the early days of genome sequencing. It is worth noting that evolutionary conservation is the single feature that reliably leads to the prediction of functionally consequential variation [48,49]. Given the continuous innovation of PPI detection techniques, we believe that a phylogenetically informed approach is timely, worthwhile and achievable.

## Current challenges

### There is a poor overlap between PPI databases

Multiple efforts have been made to curate experimentally tested PPIs from the literature and make them accessible through online databases. However, the number of functionally obligate interactions is expected to be small compared with the number of potential mis-interactions [4 9]. Due to the labour-intensive nature of experimental interaction studies, these databases can only capture a small fraction of PPIs. Therefore, several attempts have been made to predict PPIs *in silico*.

Available *in silico* prediction tools for PPIs may use a limited number of curated data sources (e.g., STRING) as training and test datasets [9,50,51]. The assumption is that PPIs with experimental validations are reliable and can be sourced from one of the largest public databases (Table 1). To test this assumption, we analysed eight popular PPI databases and found a low overlap between PPI databases (Figure 1A). No database captures a core set of interactions; conversely, most databases have a large proportion of PPIs that are unique (Figure 1A and B). Of the eight PPI databases, only 4,425 PPIs were found across five databases (Figure 1A). STRING is very inclusive, whereas SIGNOR and DIP have very little overlap with other databases (Figure 1A and B). Quantifying overlap coefficients between the PPI databases shows the median overlap coefficient of 0.35, ranging from a minimum of 0.01 (BioPlex vs SIGNOR) to a maximum of 1.0 (IntAct vs MINT, where MINT represents a smaller subset of IntAct, comprising 11% of its entries) (Figure 1A and B). Theoretically, there should be some consistency, as PPI databases all source data from published literature (except for BioPlex, Table 1). Low overlap among PPI databases hampers researchers from accurately capturing the full landscape of reliable PPIs. This fragmentation may lead to inconsistent conclusions about protein functionality and its contribution to cellular/organism fitness.

**Table 1:** Datasets of protein–protein interactions (PPIs)

Database	Dataset description	Reference
BioGRID	Physical multi-validated interactions	[34]
DIP	Experimentally determined interactions between proteins	[35]
IntAct	Experimentally derived interaction data available in the published scientific literature and from direct scientists’ submissions prior to publication in a peer-reviewed journal.	[35,36]
mentha	Source databases: MINT, IntAct, DIP, MatrixDB, BioGRID	[37]
MINT	Experimentally verified PPIs mined from the scientific literature by expert curators	[38]
SIGNOR	Experimental evidence about causal interactions between proteins and other entities of biological relevance	[40]
BioPlex	Protein interactions in human cells via affinity-purification mass spectrometry	[52]
STRING	Experimentally determined, physical-only interactions between proteins	[39]

One possible cause of low overlap between databases is the inconsistent use of IDs between databases, e.g., for protein isoforms. To investigate this further, we clustered PPI sequences using sequence similarity (MMSeq2) [53]. The results show that sequence clustering did not greatly increase the overlap between databases (Figure 1C). The difference between the overlap coefficients before and after sequence clustering was lower than 5%, confirming the inconsistency between the databases. There are at least two possibilities: (i) databases capture only a fraction of PPIs reported in the literature—an issue that was identified a decade ago [37] that persists to this day (Figure 1), and (ii) while databases may have varying inclusion criteria, which could explain some discrepancies between them, we also observe that the proteins in the databases are heavily concentrated on/predominantly belong to model organisms (Figure 1C). To address potential bias derived from model species, analyses of evolutionary conservation of PPIs may have the potential to discriminate between bona fide functional and mis-interactions. Our findings reveal several limitations of the contents of PPI databases, which hinder our ability to accurately reconstruct and understand PPI networks.

### Biased sampling of model organisms confounds the identification of conserved PPIs

Next, to explore the factors that might explain the poor overlap, we group the PPI datasets by organisms. We find that many PPIs have only been discovered in a single species, and that this distribution largely reflects the biased sampling towards a few diverse model organisms (e.g., human, yeast, fruit fly; Figure 2A and B). This shows an unintended consequence of resource allocation to a limited number of model organisms, despite the advantages gained through studying model organisms [54]. The limited number of model organisms studied hinders efforts to distinguish between non-functional, transient interactions and robustly identified functional interactions that likely have an impact on phenotype or fitness [4 6]. This sampling bias also limits our understanding of the roles of species-specific and conserved PPIs across diverse lineages.

To investigate the importance of PPI conservation, we examined the proportion of evolutionarily conserved PPIs with corresponding solved tertiary structures deposited in the Protein Data Bank (PDB). Our expectation is that the PDB provides a ‘gold standard’ of the most reliable PPIs identified to date. For each PPI, we identified homologous groups using OrthoDB annotations. This allowed us to determine the number of species across which each conserved PPI was observed. We then calculated the proportion of PPIs within each species span that have at least one corresponding complex structure deposited in the PDB.

We find that the complexes with PPIs conserved across four or more species are more likely to be structurally solved (Figure 2C, odds ratios above the points). Structurally solved PPIs are mostly ribosomal proteins, whereas structurally unresolved PPIs are mostly involved in metabolic pathways (Supplementary Figure S1). As conserved PPIs are less likely to remain unsolved (Figure 2C), this presents an attractive opportunity for solving their interactions through structural biology and *in silico* methods. This suggests that prioritising structurally unsolved, evolutionarily conserved PPIs could address gaps in functional PPI networks.

## Proposed solutions

### Most conserved PPIs remain to have structures solved

To bridge existing PPI databases with orthology-based inference frameworks, we highlighted the 10 largest conserved PPI networks to identify opportunities for resolving structurally unsolved interactions (Figure 3). The two largest interaction hubs are ribosomes and/or proteasomes, followed by the translation initiation complex (eIF3), spliceosome and respiratory complex I (NADH-ubiquinone oxidoreductase). The complete list of PPIs that remain to be structurally solved is available on GitHub (https://github.com/lcscs12345/ppi_conservation/blob/main/data/wnt_ror/unsolved.csv).

### Case Study: tackling WNT–ROR interaction through a phylogenetically informed approach

To bridge existing PPI databases with orthology-based inference frameworks, we highlighted the 10 largest conserved PPI networks to identify opportunities for resolving structurally unsolved interactions (Figure 3). The two largest interaction hubs are ribosomes and/or proteasomes, followed by the translation initiation complex (eIF3), spliceosome and respiratory complex I (NADH-ubiquinone oxidoreductase). The complete list of PPIs that remain to be structurally solved is available on GitHub (https://github.com/lcscs12345/ppi_conservation/blob/main/data/wnt_ror/unsolved.csv).

**Figure 4:**
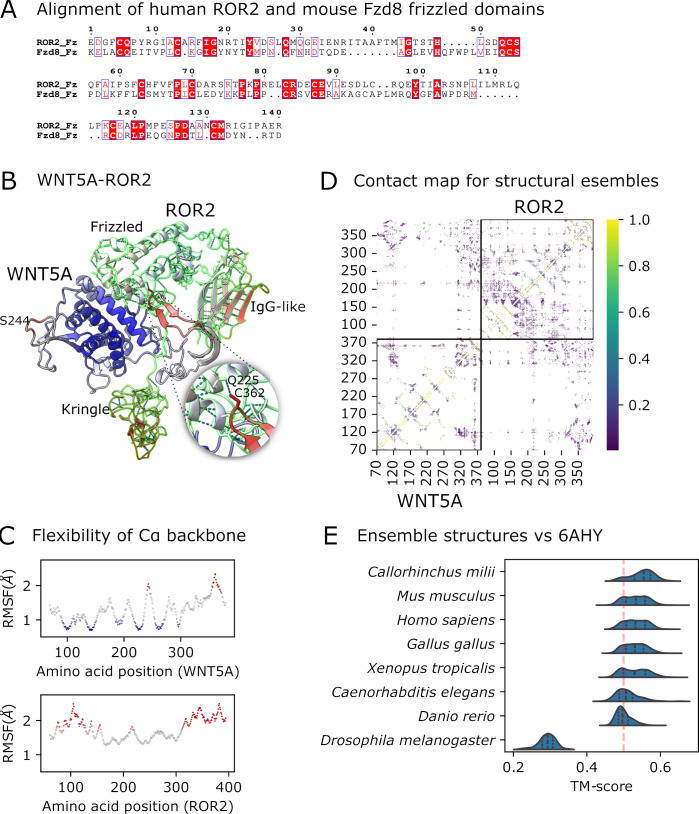
Prediction of WNT5A and ROR ligand-receptor interaction using ESMFlow. **(A**) Sequence alignment of the frizzled domains for human ROR2 and mouse Fzd8. (**B**) ESMFlow prediction of WNT5A (grey outline) ‘clamped’ on ROR2 (green outline) as shown by a representative structure from the ensemble. ROR2 consists of IgG-like, frizzled and kringle extracellular domains and a pseudokinase domain (not shown). (**C**) Cα root-mean-square fluctuations (RMSF) show that the WNT5A and ROR2 binding sites are highly flexible (black dotted circles, residues in bold; as colour-coded in the RMSF plots). See also the interaction sites annotated in the NCBI CDD entries cd19347 and cd07468. (**D**) The contact map shows the transient contacts between the WNT5A C-terminus and the ROR2 frizzled and kringle domains. The colour bar represents the frequency of contacts across the ensemble. The space outside the square boundaries corresponds to all possible spots for intermolecular contacts. (**E**) Prediction of the ensemble structures for WNT5A/B–ROR1/2 across eight species. The predicted WNT–ROR structures and the WNT3–Fzd8 frizzled domain crystal structure (PDB: 6AHY) are mostly in the same fold (template modelling [TM]-scores > 0.5, red dotted line).

Among the conserved PPIs with no available structural data, the WNT–ROR (receptor tyrosine kinase-like orphan receptor) interaction stood out as a biologically important yet unresolved case. This case study demonstrates how a phylogenetically informed approach can prioritise such interactions for computational modelling and future experimental validation. The Wnt signalling network contains a high proportion of structurally unsolved PPIs that are highly conserved (Figure 3, 5 red edges vs 3 blue edges). This includes the WNT and ROR ligand-receptor interactions previously detected across five species [55 61]. WNT is a large family of glycoproteins (OrthoDB ID: 2,874,082at2759), whereas ROR belongs to a large group of ABL proto-oncogene 1, non-receptor tyrosine kinases (OrthoDB ID: 1,614,410at2759). To understand why WNT–ROR has not been structurally solved, we searched PDB for relevant complexes and found a crystal structure for WNT3 and Fzd8 frizzled domain (PDB ID: 6AHY, human WNT3 in complex with the mouse Fzd8 frizzled domain). This is relevant to WNT–ROR, as ROR2 also has a frizzled domain that has 34% similarity with the mouse Fzd8 frizzled domain (Figure 4A). These two interactions are functionally relevant, as Wnt5A–ROR1/2 interactions inhibit Wnt3 signalling in *Caenorhabditis elegans* [62].

The crystal structure 6AHY shows an interaction involving O-palmitoleoyl Ser 244 of WNT5A (or Ser 223 of WNT5B). However, a recently solved ROR2 crystal structure lacks a hydrophobic groove to accommodate the palmitoleate moiety (PDB ID: 9FSE) [60]. This study proposed that WNT–ROR ligand-receptor interaction is weak but highly specific, in which a co-receptor may be involved. Therefore, solving the WNT–ROR interaction structurally may be highly challenging. To tackle this computationally, we tested the WNT5A–ROR2 interaction using AlphaFold3 [27]. We used a palmitic acid molecule as a ligand to mimic the palmitoleate moiety, as this has been useful in predicting 6AHY accurately. 6AHY likely contributed to the AlphaFold3 training set using PDB structures released before 2021-09-30. We obtained ipTM (interface predicted template modelling) confidence scores of 0.79 for 6AHY but 0.18 for WNT5A–ROR2, indicating a low confidence in predicting the WNT5A–ROR2 interface.

We further predicted WNT5A–ROR2 interactions using ESMFlow, which produces structural ensembles of proteins without requiring multiple sequence alignments as input [63,64]. One limitation of this prediction is that post-translational modifications could not be included. Although potential interactions involving the WNT5A palmitoleate modification could not be captured, the amino acid residues around S244 were predicted to be flexible (Figure 4B and C). We also predicted transient interactions between the WNT5A C-terminus and the ROR2 frizzled and kringle domains (Figure 4D). Both the S244 and C-terminal regions of WNT5A were predicted to be highly flexible, suggesting that WNT5A ‘clamps’ on ROR2. Such transient structure could not be predicted using AlphaFold by default [65 67]. Although AlphaFold can be customised to predict alternative protein structures using subsets of multiple sequence alignments, it is a weak predictor of fold switching [65]. In addition, we predicted the structural ensembles for WNT5A/B–ROR1/2 across eight species. We found that WNT–ROR and WNT3–Fzd8 frizzled domain (PDB: 6AHY) are mostly in the same fold, except for those in *C. elegans and Drosophila melanogaster* that are more distantly related to others. (Figure 4E, 75% of predicted complexes have TM-scores > 0.5) [68]. This supports the notion that WNT–ROR interactions are highly conserved. This example highlights that key PPIs we don’t understand structurally can be tackled through a phylogenetically informed approach.

## Future directions

We have shown that the majority of PPIs are sparsely curated in well-established databases. Calderone et al. found that PPI databases were sparse in 2013 [37], and this issue persists despite cross-referencing across multiple databases and the rapid growth in computational power over the past 12 years. While meta-databases offer a temporary solution, addressing the inconsistencies between databases requires significant investment in community-driven curation efforts and sustained funding support. Limited curation can lead to inaccurate predictions of protein function, misallocation of research funds and hinder drug discovery efforts. Moreover, for machine learning purposes, a large and well-curated set of reliable PPIs is required to distinguish functional interactions from random noise.

Through our efforts in consolidating PPIs, we have found that PPIs detected across multiple species are more likely to be robustly validated by experimental structural solutions. This suggests that PPIs detected across multiple species are more reliable, while those identified in only one species may represent random interactions lacking validation of their importance for organismal fitness. This presents an attractive opportunity for structural biologists to solve core, conserved PPIs found across multiple species, as less conserved PPIs are likely to be accessory interactions. While focusing on conserved PPIs has been valuable, it is essential to acknowledge the potential importance of less conserved PPIs and strive for a more balanced research agenda with a broad perspective.

We propose creating a set of core/gold-standard PPIs using a multi-disciplinary approach combining functional genomics, computational modelling and structural biology. This phylogenetically informed approach will span across multiple species as inspired by the Zoonomia, GEBA and Protein Structure Initiative: Biology projects [43 47,69,70]. The experimental results and evolutionary models (e.g., ESM3 and EVcomplex2) may help reconstruct the evolutionary history of PPIs and identify functionally important amino acid residues [28,71]. For example, building upon successful coevolutionary analyses in *S. cerevisiae* [72], we propose extending this approach to other species to distinguish conserved core interactions from evolutionarily transient ones and resolve inconsistencies between existing PPI databases. Specifically, inclusion in the ‘gold standard’ will require evidence from at least two independent sources (e.g., experimental validation, a strong coevolutionary signal and consistent prediction across multiple computational methods) and demonstrated conservation across diverse species. For non-model organisms, we suggest prioritising PPIs with high-confidence computational predictions (e.g., TM-scores > 0.5 and ipTM scores > 0.6) for experimental validation [73], particularly in underrepresented clades such as lophotrochozoans and early branching bilaterians. Community input and expert curation should be integral to the ongoing maintenance and expansion of this resource.

We envisage that the proposed high-confidence, well-curated PPI dataset will serve as a foundational resource for the scientific community. It will facilitate the development of more accurate computational methods and experimental designs, and provide a reliable framework for studying protein function, disease mechanisms and drug discovery. We encourage researchers to contribute to this effort by sharing data, expertise and computational tools, and we advocate for sustained funding to support the critical work of PPI curation.

**Perspectives**

- There is limited overlap among protein–protein interaction (PPI) databases, which hinders the identification and exploration of the reliable PPI landscape.
- The focus on model organisms limits the identification of conserved PPIs across diverse species.
- Many conserved PPIs lack structurally solved complexes, providing an opportunity for structural and computational biologists to address gaps. Evolutionary models of PPI conservation may help distinguish between conserved and evolutionarily transient interactions.

## Supplementary material

online supplementary material 1.

## Open Access

This article has been published open access under our Subscribe to Open programme, made possible through the support of our subscribing institutions, learn more here: https://portlandpress.com/pages/open_access_options_and_prices#conditional

## Abbreviations

GEBA: Genomic Encyclopedia of Bacteria and Archaea
PDB: Protein Data Bank
PPI: protein–protein interaction
RMSF: root-mean-square fluctuation
ROR: receptor tyrosine kinase-like orphan receptor
ipTM: interface predicted template modelling

## References

[1] Lu H. Zhou Q. He J. Jiang Z. Peng C. Tong R. et al. 2020 Recent advances in the development of protein-protein interactions modulators: mechanisms and clinical trials Signal Transduct. Target. Ther. 5 213 10.1038/s41392-020-00315-3 32968059 PMC7511340

[2] Cheng F. Zhao J. Wang Y. Lu W. Liu Z. Zhou Y. et al. 2021 Comprehensive characterization of protein-protein interactions perturbed by disease mutations Nat. Genet. 53 342 353 10.1038/s41588-020-00774-y 33558758 PMC8237108

[3] Peng X. Wang J. Peng W. Wu F.X 2016 Briefings Protein–protein interactions: detection, reliability assessment and applications https://academic.oup.com/bib/article-abstract/18/5/798/2562794

[4] Schulz L. Sendker F.L. Hochberg G.K.A 2022 Non-adaptive complexity and biochemical function Curr. Opin. Struct. Biol. 73 102339 10.1016/j.sbi.2022.102339 35247750

[5] Abrusán G. Foguet C 2023 An assessment of quaternary structure functionality in homomer protein complexes Mol. Biol. Evol. 40 msad070 10.1093/molbev/msad070 36947103 PMC10118308

[6] Brunet T.D.P. Doolittle W.F 2018 The generality of constructive neutral evolution Biol. Philos. 33 10.1007/s10539-018-9614-6

[7] Fischer L. Rappsilber J 2024 Rescuing error control in crosslinking mass spectrometry Mol. Syst. Biol. 20 1076 1084 10.1038/s44320-024-00057-2 39095427 PMC11368935

[8] Richards A.L. Eckhardt M. Krogan N.J 2021 Mass spectrometry-based protein-protein interaction networks for the study of human diseases Mol. Syst. Biol. 17 e8792 10.15252/msb.20188792 33434350 PMC7803364

[9] Blumenthal D.B. Lucchetta M. Kleist L. Fekete S.P. List M. Schaefer M.H 2024 Emergence of power law distributions in protein-protein interaction networks through study bias Elife 13 e99951 10.7554/eLife.99951 39660719 PMC11718653

[10] Grassmann G. Miotto M. Desantis F. Di Rienzo L. Tartaglia G.G. Pastore A. et al. 2024 Computational approaches to predict protein-protein interactions in crowded cellular environments Chem. Rev. 124 3932 3977 10.1021/acs.chemrev.3c00550 38535831 PMC11009965

[11] Danielsson J. Mu X. Lang L. Wang H. Binolfi A. Theillet F.-X. et al. 2015 Thermodynamics of protein destabilization in live cells Proc. Natl. Acad. Sci. U.S.A. 112 12402 12407 10.1073/pnas.1511308112 26392565 PMC4603463

[12] Ando T. Yu I. Feig M. Sugita Y 2016 Thermodynamics of Macromolecular Association in Heterogeneous Crowding Environments: Theoretical and Simulation Studies with a Simplified Model J. Phys. Chem. B 120 11856 11865 10.1021/acs.jpcb.6b06243 27797534 PMC8054318

[13] Nooren I.M.A. Thornton J.M 2003 Diversity of protein-protein interactions EMBO J. 22 3486 3492 10.1093/emboj/cdg359 12853464 PMC165629

[14] Veale C.G.L. Clarke D.J 2024 Mass spectrometry-based methods for characterizing transient protein–protein interactions Trends in Chemistry 6 377 391 10.1016/j.trechm.2024.05.002

[15] Ide N.A. Gentry R.C. Rudbach M.A. Yoo K. Velez P.K. Comunale V.M. et al. 2024 A dynamic compositional equilibrium governs mRNA recognition by eIF3 bioRxiv 2024.04.25.581977 10.1101/2024.04.25.581977 38712078 PMC11071631

[16] Beusch I. Madhani H.D 2024 Understanding the dynamic design of the spliceosome Trends Biochem. Sci. 49 583 595 10.1016/j.tibs.2024.03.012 38641465

[17] Hochberg G.K.A. Liu Y. Marklund E.G. Metzger B.P.H. Laganowsky A. Thornton J.W 2020 A hydrophobic ratchet entrenches molecular complexes Nature 588 503 508 10.1038/s41586-020-3021-2 33299178 PMC8168016

[18] Michel F. Crucifix C. Granger F. Eiler S. Mouscadet J.-F. Korolev S. et al. 2009 Structural basis for HIV-1 DNA integration in the human genome, role of the LEDGF/P75 cofactor EMBO J. 28 980 991 10.1038/emboj.2009.41 19229293 PMC2670869

[19] Maillot B. Lévy N. Eiler S. Crucifix C. Granger F. Richert L. et al. 2013 Structural and functional role of INI1 and LEDGF in the HIV-1 preintegration complex PLoS ONE 8 e60734 10.1371/journal.pone.0060734 23593299 PMC3623958

[20] Levy N. Eiler S. Pradeau-Aubreton K. Maillot B. Stricher F. Ruff M 2016 Production of unstable proteins through the formation of stable core complexes Nat. Commun. 7 10932 10.1038/ncomms10932 26983699 PMC4800440

[21] Xing S. Wallmeroth N. Berendzen K.W. Grefen C 2016 Techniques for the analysis of protein-protein interactions i*n Vivo* Plant Physiol. 171 727 758 10.1104/pp.16.00470 27208310 PMC4902627

[22] Rao V.S. Srinivas K. Sujini G.N. Kumar G.N.S 2014 Protein-protein interaction detection: methods and analysis Int. J. Proteomics 2014 147648 10.1155/2014/147648 24693427 PMC3947875

[23] Fredriksson S. Gullberg M. Jarvius J. Olsson C. Pietras K. Gústafsdóttir S.M. et al. 2002 Protein detection using proximity-dependent DNA ligation assays Nat. Biotechnol. 20 473 477 10.1038/nbt0502-473 11981560

[24] Wang P. Yang Y. Hong T. Zhu G 2021 Proximity ligation assay: an ultrasensitive method for protein quantification and its applications in pathogen detection Appl. Microbiol. Biotechnol. 105 923 935 10.1007/s00253-020-11049-1 33427935

[25] Fields S. Song O 1989 A novel genetic system to detect protein-protein interactions Nature 340 245 246 10.1038/340245a0 2547163

[26] Kaboord B. Perr M 2008 Isolation of proteins and protein complexes by immunoprecipitation Methods Mol. Biol. 424 349 364 10.1007/978-1-60327-064-9_27 18369874

[27] Abramson J. Adler J. Dunger J. Evans R. Green T. Pritzel A. et al. 2024 Accurate structure prediction of biomolecular interactions with AlphaFold 3 Nature 630 493 500 10.1038/s41586-024-07487-w 38718835 PMC11168924

[28] Hayes T. Rao R. Akin H. Sofroniew N.J. Oktay D. Lin Z. et al. 2024 Simulating 500 million years of evolution with a language model Synthetic Biology 10.1101/2024.07.01.600583 39818825

[29] Salgania H.K. Metz J. Jeske M 2024 ReLo is a simple and rapid colocalization assay to identify and characterize direct protein-protein interactions Nat. Commun. 15 2875 10.1038/s41467-024-47233-4 38570497 PMC10991417

[30] Reed T.J. Tyl M.D. Tadych A. Troyanskaya O.G. Cristea I.M 2024 Tapioca: a platform for predicting de novo protein-protein interactions in dynamic contexts Nat. Methods 21 488 500 10.1038/s41592-024-02179-9 38361019 PMC11249048

[31] Janakaloti Narayanareddy B.R. Allipeta N.R. Allard J. Gross S.P 2024 A new method to experimentally quantify dynamics of initial protein-protein interactions Commun. Biol. 7 311 10.1038/s42003-024-05914-2 38472292 PMC10933273

[32] Lazarewicz N. Le Dez G. Cerjani R. Runeshaw L. Meurer M. Knop M. et al. 2024 Accurate and sensitive interactome profiling using a quantitative protein-fragment complementation assay Cell Rep. Methods 4 100880 10.1016/j.crmeth.2024.100880 39437715 PMC11573789

[33] Tang H.-W. Spirohn K. Hu Y. Hao T. Kovács I.A. Gao Y. et al. 2023 Next-generation large-scale binary protein interaction network for Drosophila melanogaster Nat. Commun. 14 2162 10.1038/s41467-023-37876-0 37061542 PMC10105736

[34] Oughtred R. Rust J. Chang C. Breitkreutz B.-J. Stark C. Willems A. et al. 2021 The BioGRID database: A comprehensive biomedical resource of curated protein, genetic, and chemical interactions Protein Sci. 30 187 200 10.1002/pro.3978 33070389 PMC7737760

[35] Xenarios I. Salwínski L. Duan X.J. Higney P. Kim S.-M. Eisenberg D 2002 DIP, the Database of Interacting Proteins: a research tool for studying cellular networks of protein interactions Nucleic Acids Res. 30 303 305 10.1093/nar/30.1.303 11752321 PMC99070

[36] Del Toro N. Shrivastava A. Ragueneau E. Meldal B. Combe C. Barrera E. et al. 2022 The IntAct database: efficient access to fine-grained molecular interaction data Nucleic Acids Res. 50 D648 D653 10.1093/nar/gkab1006 34761267 PMC8728211

[37] Calderone A. Castagnoli L. Cesareni G 2013 mentha: a resource for browsing integrated protein-interaction networks Nat. Methods 10 690 691 10.1038/nmeth.2561 23900247

[38] Calderone A. Iannuccelli M. Peluso D. Licata L 2020 Using the MINT Database to Search Protein Interactions Curr. Protoc. Bioinformatics 69 e93 10.1002/cpbi.93 31945268

[39] Szklarczyk D. Kirsch R. Koutrouli M. Nastou K. Mehryary F. Hachilif R. et al. 2023 The STRING database in 2023: protein-protein association networks and functional enrichment analyses for any sequenced genome of interest Nucleic Acids Res. 51 D638 D646 10.1093/nar/gkac1000 36370105 PMC9825434

[40] Lo Surdo P. Iannuccelli M. Contino S. Castagnoli L. Licata L. Cesareni G. et al. 2023 SIGNOR 3.0, the SIGnaling network open resource 3.0: 2022 update Nucleic Acids Res. 51 D631 D637 10.1093/nar/gkac883 36243968 PMC9825604

[41] López Y. Nakai K. Patil A 2015 HitPredict version 4: comprehensive reliability scoring of physical protein-protein interactions from more than 100 species Database (Oxford) 2015 bav117 10.1093/database/bav117 26708988 PMC4691340

[42] Kamburov A. Herwig R 2022 ConsensusPathDB 2022: molecular interactions update as a resource for network biology Nucleic Acids Res. 50 D587 D595 10.1093/nar/gkab1128 34850110 PMC8728246

[43] Wu D. Hugenholtz P. Mavromatis K. Pukall R. Dalin E. Ivanova N.N. et al. 2009 A phylogeny-driven genomic encyclopaedia of Bacteria and Archaea Nature 462 1056 1060 10.1038/nature08656 20033048 PMC3073058

[44] Kyrpides N.C. Hugenholtz P. Eisen J.A. Woyke T. Göker M. Parker C.T. et al. 2014 Genomic encyclopedia of bacteria and archaea: sequencing a myriad of type strains PLoS Biol. 12 e1001920 10.1371/journal.pbio.1001920 25093819 PMC4122341

[45] Mukherjee S. Seshadri R. Varghese N.J. Eloe-Fadrosh E.A. Meier-Kolthoff J.P. Göker M. et al. 2017 1,003 reference genomes of bacterial and archaeal isolates expand coverage of the tree of life Nat. Biotechnol. 35 676 683 10.1038/nbt.3886 28604660

[46] Kirilenko B.M. Munegowda C. Osipova E. Jebb D. Sharma V. Blumer M. et al. 2023 Integrating gene annotation with orthology inference at scale Science 380 eabn3107 10.1126/science.abn3107 37104600 PMC10193443

[47] Christmas M.J. Kaplow I.M. Genereux D.P. Dong M.X. Hughes G.M. Li X. et al. 2023 Evolutionary constraint and innovation across hundreds of placental mammals Science 380 eabn3943 10.1126/science.abn3943 37104599 PMC10250106

[48] Eddy S.R 2005 A model of the statistical power of comparative genome sequence analysis PLoS Biol. 3 e10 10.1371/journal.pbio.0030010 15660152 PMC539325

[49] Cooper G.M. Brudno M. Green E.D. Batzoglou S. Sidow A. NISC Comparative Sequencing Program 2003 Quantitative estimates of sequence divergence for comparative analyses of mammalian genomes Genome Res. 13 813 820 10.1101/gr.1064503 12727901 PMC430923

[50] Soleymani F. Paquet E. Viktor H. Michalowski W. Spinello D 2022 Protein–protein interaction prediction with deep learning: a comprehensive review Comput. Struct. Biotechnol. J. 20 5316 5341 10.1016/j.csbj.2022.08.070 36212542 PMC9520216

[51] Kewalramani N. Emili A. Crovella M 2023 State-of-the-art computational methods to predict protein-protein interactions with high accuracy and coverage Proteomics 23 e2200292 10.1002/pmic.202200292 37401192

[52] Huttlin E.L. Bruckner R.J. Navarrete-Perea J. Cannon J.R. Baltier K. Gebreab F et al. 2021 Dual proteome-scale networks reveal cell-specific remodeling of the human interactome Cell 184 3022 3040 10.1016/j.cell.2021.04.011 33961781 PMC8165030

[53] Steinegger M. Söding J 2017 MMseqs2 enables sensitive protein sequence searching for the analysis of massive data sets Nat. Biotechnol. 35 1026 1028 10.1038/nbt.3988 29035372

[54] Müller B. Grossniklaus U 2010 Model organisms--A historical perspective J. Proteomics 73 2054 2063 10.1016/j.jprot.2010.08.002 20727995

[55] Ripp C. Loth J. Petrova I. Linnemannstöns K. Ulepic M. Fradkin L et al. 2018 Ror is a nervous system-specific co-receptor for Wnt ligands Biol. Open 7 10.1242/bio.033001 PMC6262871 30341100

[56] Mikels A.J. Nusse R 2006 Purified Wnt5a protein activates or inhibits beta-catenin-TCF signaling depending on receptor context PLoS Biol. 4 e115 10.1371/journal.pbio.0040115 16602827 PMC1420652

[57] Oishi I. Suzuki H. Onishi N. Takada R. Kani S. Ohkawara B. et al. 2003 The receptor tyrosine kinase Ror2 is involved in non‐canonical Wnt5a/JNK signalling pathway Genes Cells 8 645 654 10.1046/j.1365-2443.2003.00662.x 12839624

[58] Green J.L. Inoue T. Sternberg P.W 2007 The *C. elegans* ROR receptor tyrosine kinase, CAM-1,non-autonomously inhibits the Wnt pathway . Development (Rome) 134 4053 4062 10.1242/dev.005363 17942487

[59] Wang J. Ding M 2018 Robo and Ror function in a common receptor complex to regulate Wnt-mediated neurite outgrowth in *Caenorhabditis elegans* . Proc. Natl. Acad. Sci. U.S.A 115 E2254 E2263 10.1073/pnas.1717468115 29463707 PMC5877952

[60] Griffiths S.C. Tan J. Wagner A. Blazer L.L. Adams J.J. Srinivasan S. et al. 2024 Structure and function of the ROR2 cysteine-rich domain in vertebrate noncanonical WNT5A signaling Elife 13 e71980 10.7554/eLife.71980 38780011 PMC11219042

[61] Peradziryi H. Kaplan N.A. Podleschny M. Liu X. Wehner P. Borchers A. et al. 2011 PTK7/Otk interacts with Wnts and inhibits canonical Wnt signalling EMBO J. 30 3729 3740 10.1038/emboj.2011.236 21772251 PMC3173783

[62] Bainbridge T.W. DeAlmeida V.I. Izrael-Tomasevic A. Chalouni C. Pan B. Goldsmith J. et al. 2014 Evolutionary divergence in the catalytic activity of the CAM-1, ROR1 and ROR2 kinase domains PLoS ONE 9 e102695 10.1371/journal.pone.0102695 25029443 PMC4100928

[63] Jing B. Berger B. Jaakkola T 2024 AlphaFold meets flow matching for generating protein ensembles arXiv [q-bio.BM] 10.48550/ARXIV.2402.04845

[64] GitHub GitHub - lcscs12345/alphaflow: AlphaFold Meets Flow Matching for Generating Protein Ensembles https://github.com/lcscs12345/alphaflow

[65] Chakravarty D. Schafer J.W. Chen E.A. Thole J.F. Ronish L.A. Lee M. et al. 2024 AlphaFold predictions of fold-switched conformations are driven by structure memorization Nat. Commun. 15 7296 10.1038/s41467-024-51801-z 39181864 PMC11344769

[66] Wayment-Steele H.K. Ojoawo A. Otten R. Apitz J.M. Pitsawong W. Hömberger M. et al. 2024 Predicting multiple conformations via sequence clustering and AlphaFold2 Nature 625 832 839 10.1038/s41586-023-06832-9 37956700 PMC10808063

[67] Monteiro da Silva G. Cui J.Y. Dalgarno D.C. Lisi G.P. Rubenstein B.M 2024 High-throughput prediction of protein conformational distributions with subsampled AlphaFold2 Nat. Commun. 15 2464 10.1038/s41467-024-46715-9 38538622 PMC10973385

[68] Xu J. Zhang Y 2010 How significant is a protein structure similarity with TM-score = 0.5? Bioinformatics 26 889 895 10.1093/bioinformatics/btq066 20164152 PMC2913670

[69] Berman H.M. Westbrook J.D. Gabanyi M.J. Tao W. Shah R. Kouranov A. et al. 2009 The protein structure initiative structural genomics knowledgebase Nucleic Acids Res. 37 D365 8 10.1093/nar/gkn790 19010965 PMC2686438

[70] Cormier C.Y. Mohr S.E. Zuo D. Hu Y. Rolfs A. Kramer J. et al. 2010 Protein Structure Initiative Material Repository: an open shared public resource of structural genomics plasmids for the biological community Nucleic Acids Res. 38 D743 9 10.1093/nar/gkp999 19906724 PMC2808882

[71] Green A.G. Elhabashy H. Brock K.P. Maddamsetti R. Kohlbacher O. Marks D.S 2021 Large-scale discovery of protein interactions at residue resolution using co-evolution calculated from genomic sequences Nat. Commun. 12 1396 10.1038/s41467-021-21636-z 33654096 PMC7925567

[72] Humphreys I.R. Pei J. Baek M. Krishnakumar A. Anishchenko I. Ovchinnikov S. et al. 2021 Computed structures of core eukaryotic protein complexes Science 374 eabm4805 10.1126/science.abm4805 34762488 PMC7612107

[73] EMBL-EBI Confidence scores in AlphaFold-Multimer https://www.ebi.ac.uk/training/online/courses/alphafold/inputs-and-outputs/evaluating-alphafolds-predicted-structures-using-confidence-scores/confidence-scores-in-alphafold-multimer/

