# Supplementary material for: Towards a phylogenetically informed approach to solving protein–protein interactions
Source: Biochem Soc Trans. 2025 Dec 3;53(6):BST20253031. doi: 10.1042/BST20253031 (PMC12751078; doi:10.1042/BST20253031)
Supplement: online supplementary material 1. [file BST-53-06-BST20253031-s001.pdf]

Supplementary Notes

Data

Datasets were obtained from eight PPI databases (Table 1). These include BioGRID 4.4.204 [1], DIP 20170205 [2], IntAct 2021-10-13 [2,3], mentha 2021-12-20 [4], MINT 2021-12-19 [5], SIGNOR Jan2022 [6], BioPlex 3.0 [7], and STRING 11.5 [8]. For BioGRID and STRING, physical-only PPIs were available as separate files for download. These files were retrieved as indirect or functional interactions are out of the scope of this study. To ensure reliability, only experimentally validated datasets were selected for analysis. Datasets were parsed using Pandas v2.0.3 [9] and pandarallel v1.6.4 [10] into data frames.

ID mapping

STRING uses mainly ENSEMBL IDs, whereas other datasets use a mix of UniProt primary and secondary accessions and NCBI RefSeq accessions. To systematically compare these datasets, ID mapping was performed as follows: For STRING, protein IDs were first mapped to UniProt accessions based on protein alias cross-referencing in STRING (protein.aliases.UniProt_AC.v11.5.txt.gz). To further unify the accession numbers across all the datasets by UniProt primary accessions, the UniProt ID mapping web service was used to retrieve UniProt primary accessions, NCBI taxonomy IDs, OrthoDB IDs [11], PDB IDs [12], and KEGG pathways IDs [13]. However, the OrthoDB group names and KEGG pathway names were not available through the UniProt ID mapping web service. Therefore, the OrthoDB group names and KEGG pathway names were retrieved from their respective databases using the IDs.

Overlap coefficients

To visualise the consistency between PPI databases, the data frames were merged by UniProt primary ID pairs. The overlap among the databases was plotted using UpSetPlot v0.8.0 [14] (Fig 1A and 2A).

The inconsistent use of IDs between PPI databases may result in low overlap between the databases (Fig 1A and B). To test this possibility, MMseqs2 [15] was used for sequence clustering using different identity thresholds (Fig 1B, right panel). Protein pairs were grouped by sequence cluster IDs instead of relying on UniProt IDs. To quantify the extent of overlap between PPI databases, overlap coefficients were calculated using a custom Python function with NumPy v1.24.3 [16] as follows:

$$Overlap(X, Y) = \frac{|X \cap Y|}{min(|X|, |Y|)}$$

To visualise overlap coefficients, a heatmap was plotted (Fig 1B, right panel). Changes in overlap coefficients before and after sequence clustering were plotted as a strip plot (Fig 1B, left panel). These plots were created using seaborn v0.12.2 [17] and Matplotlib v3.7.2 [18].

PPI conservation

PPIs were considered conserved if they were found in more than one species, i.e. the presence of homologous pairs of sequences in any PPI databases. To estimate the conservation of PPIs, UniProt ID pairs were first mapped to OrthoDB ID pairs. The overlap of homologous protein pairs (OrthoDB ID pairs) across species (NCBI taxonomy IDs) was visualised as an upset plot (Fig 2A). To compare the number of conserved PPIs across databases, the number of OrthoDB ID pairs corresponding to one species or multiple species was calculated (Fig 2B).

To determine whether conserved PPIs are likely to be structurally solved, the number of OrthoDB ID pairs with corresponding PDB structures was calculated and the percentages of unsolved complexes were derived (Fig 2C). To highlight the opportunities for tackling conserved PPIs that have yet to be structurally solved, PPI networks were constructed and plotted using NetworkX v.3.1 [19] and Netgraph v4.13.2 [20], respectively (Fig 3). To identify large interaction hubs (nodes for the PPI networks), conserved PPIs (OrthoDB IDs) were mapped to their corresponding KEGG pathways (Fig 3). The complete list of PPIs lacking structural information is available on GitHub (https://github.com/lcscs12345/ppi_conservation/blob/main/data/unsolved.csv).

Protein complex prediction

To predict WNT-ROR interactions, the sequences were retrieved from UniProt by querying “WNT5A”, “WNT5B”, “ROR1”, and “ROR2”. The sequences for *C. elegans* were retrieved through the ID mapping service separately as they have different protein names. The distribution of these proteins across species was examined by grouping them by the taxonomic rank class.

As ROR receptors have multiple protein domains, the completeness of ROR sequences was examined by (i) retrieving their protein domain annotations through the InterPro REST API [21] and (ii) hmmsearch against the Pfam 36.0 models (HMMER3 v3.4) [22–24]. Only ROR2 sequences containing all four domains (IgG-like, frizzled, and kringle extracellular domains and a pseudokinase domain) with at least 20% coverage are selected. The WNT and ROR sequences for *Homo sapiens*, *Mus musculus*, *Danio rerio*, *Xenopus tropicalis*, *Gallus gallus*, *Callorhinchus milii*, *C. elegans*, and *D. melanogaster* were selected for protein complex prediction.

To identify structurally similar complexes, the keywords “WNT”, “ROR”, and “Frizzled” were used to query the PDB database. The PDB structure of a relevant complex (PDB ID: 6AHY) was downloaded. This is a crystal structure for human WNT3 and the mouse Fzd8 frizzled domain. To compare the sequence similarity between the human ROR2 and mouse Fzd8, the amino acid sequences were aligned using MAFFT v7 [25] and visualised using ESPript v3.0 [26] (Fig 4A).

ESMFlow was initially designed to predict structural ensembles of single-subunit proteins [27]. To enable protein complex prediction, we modified ESMFlow to extend its capability to simulate the dynamics of protein complexes [28]. A linker of 25 glycine residues (represented as “:”) was inserted between the concatenated WNT and ROR sequences

(WNT-$[G]_{25}$-ROR, as WNT and the N-terminal region of ROR are located extracellularly) [29]. ESMFlow was modified [28], such that the PDB output files can be parsed into multi-chain structures using BioPandas v0.5.1 [30]. To enable reproducible analysis, an Apptainer image [31,32] was created for ESMFlow with dependencies installed [33]. The predicted structures were visualised using UCSC ChimeraX [34] (Fig 4B).

To determine the structural flexibility of the predicted complexes, the root-mean-square fluctuations (RMSF) for the Cα backbones were calculated using MDTraj v1.10 [35] (Fig 4C). To visualise intermolecular contacts, the contact map for the ESMFlow trajectory was plotted using Contact Map Explorer v0.7.0 [36] (Fig 4D). To evaluate structural similarity, TM-scores for the predicted WNT-ROR structures vs. the reference PDB structure 6AHY were calculated using US-align [37]. To compare the TM-scores between species, the distributions of TM-scores were plotted as violin plots (Fig 4E).

Data Availability

Jupyter notebooks [38], Slurm scripts [39] for ESMFlow and US-align, and data to reproduce the results are available on GitHub (https://github.com/lcscs12345/ppi_conservation) and Zenodo (https://doi.org/10.5281/zenodo.14020181), respectively. The list of PPIs that remain to be structurally solved is available in the GitHub repository. The custom version of AlphaFlow/ESMFlow is available on GitHub (https://github.com/lcscs12345/alphaflow) and the Apptainer image with dependencies is available on Docker Hub (https://hub.docker.com/r/lcscs12345/alphaflow).

Supplementary Figures

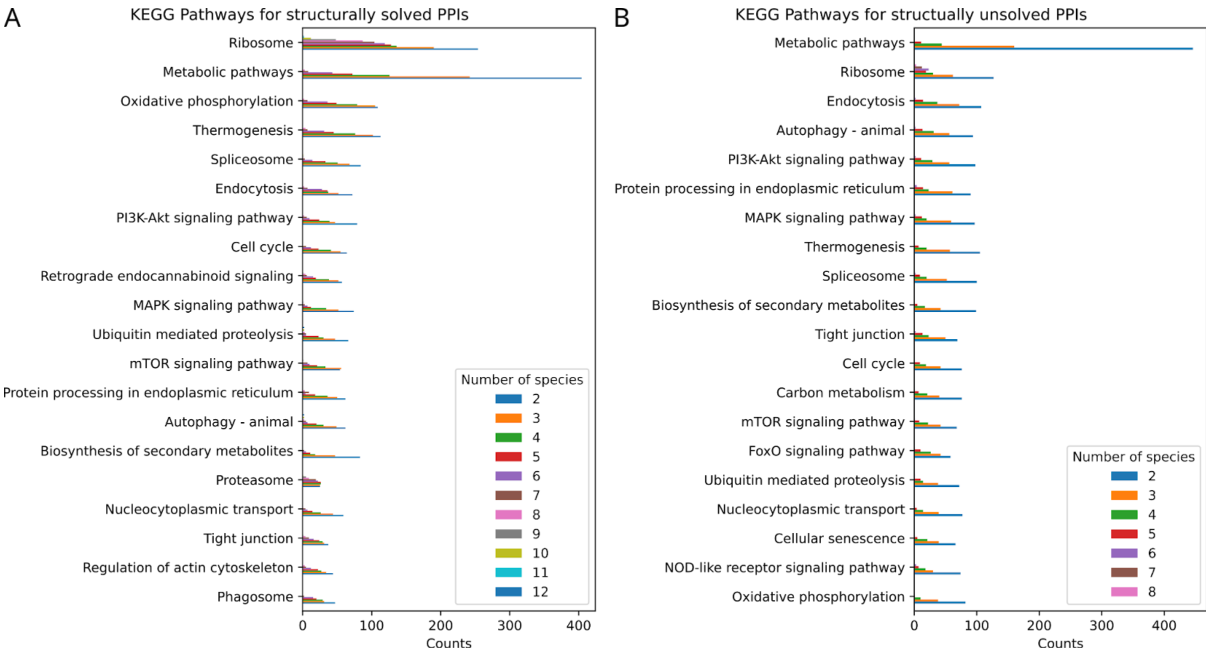

Fig S1. KEGG Pathway analysis for PPIs. Structurally solved PPIs are enriched for ribosomal proteins, whereas structurally unresolved PPIs are enriched for protein involved in metabolic pathways.

References

1 Oughtred, R., Rust, J., Chang, C., Breitkreutz, B.-J., Stark, C., Willems, A., et al. (2021) The BioGRID database: A comprehensive biomedical resource of curated protein, genetic, and chemical interactions. *Protein Sci.* **30**, 187–200

2 Xenarios, I., Salwínski, L., Duan, X.J., Higney, P., Kim, S.-M. and Eisenberg, D. (2002) DIP, the Database of Interacting Proteins: a research tool for studying cellular networks of protein interactions. *Nucleic Acids Res.* **30**, 303–305

3 Del Toro, N., Shrivastava, A., Ragueneau, E., Meldal, B., Combe, C., Barrera, E., et al. (2022) The IntAct database: efficient access to fine-grained molecular interaction data. *Nucleic Acids Res.* **50**, D648–D653

4 Calderone, A., Castagnoli, L. and Cesareni, G. (2013) mentha: a resource for browsing integrated protein-interaction networks. *Nat. Methods* **10**, 690–691

5 Calderone, A., Iannuccelli, M., Peluso, D. and Licata, L. (2020) Using the MINT Database to Search Protein Interactions. *Curr. Protoc. Bioinformatics* **69**, e93

6 Lo Surdo, P., Iannuccelli, M., Contino, S., Castagnoli, L., Licata, L., Cesareni, G., et al. (2023) SIGNOR 3.0, the SIGnaling network open resource 3.0: 2022 update. *Nucleic Acids Res.* **51**, D631–D637

7 Huttlin, E.L., Bruckner, R.J., Navarrete-Perea, J., Cannon, J.R., Baltier, K., Gebreab, F., et al. (2021) Dual proteome-scale networks reveal cell-specific remodeling of the human interactome. *Cell* **184**, 3022–3040.e28

8 Szklarczyk, D., Kirsch, R., Koutrouli, M., Nastou, K., Mehryary, F., Hachilif, R., et al. (2023) The STRING database in 2023: protein-protein association networks and functional enrichment analyses for any sequenced genome of interest. *Nucleic Acids Res.* **51**, D638–D646

9 The pandas development team (2024) pandas-dev/pandas: Pandas. Zenodo; 2024. https://doi.org/10.5281/ZENODO.3509134

10 pandarallel. PyPI. https://pypi.org/project/pandarallel/

11 Kuznetsov, D., Tegenfeldt, F., Manni, M., Seppey, M., Berkeley, M., Kriventseva, E.V., et al. (2023) OrthoDB v11: annotation of orthologs in the widest sampling of organismal diversity. *Nucleic Acids Res* **51**, D445–D451

12 Burley, S.K., Bhikadiya, C., Bi, C., Bittrich, S., Chao, H., Chen, L., et al. (2023) RCSB Protein Data Bank (RCSB.org): delivery of experimentally-determined PDB structures alongside one million computed structure models of proteins from artificial intelligence/machine learning. *Nucleic Acids Res* **51**, D488–D508

13 Kanehisa, M., Furumichi, M., Tanabe, M., Sato, Y. and Morishima, K. (2017) KEGG: new perspectives on genomes, pathways, diseases and drugs. *Nucleic Acids Res* **45**, D353–D361

14 UpSetPlot. PyPI. https://pypi.org/project/UpSetPlot/

15 Steinegger, M. and Söding, J. (2017) MMseqs2 enables sensitive protein sequence searching for the analysis of massive data sets. *Nat Biotechnol* **35**, 1026–1028

16 Harris, C.R., Millman, K.J., van der Walt, S.J., Gommers, R., Virtanen, P., Cournapeau, D., et al. (2020) Array programming with NumPy. *Nature* **585**, 357–362

17 Waskom, M. (2021) seaborn: statistical data visualization. *J. Open Source Softw.* **6**, 3021

18 Hunter, J.D. (2007) Matplotlib: A 2D Graphics Environment. *Comput. Sci. Eng.* **9**, 90–95

19 Hagberg, A., Swart, P.J. and Schult, D.A. (2008) Exploring network structure, dynamics, and function using NetworkX. Los Alamos National Laboratory (LANL), Los Alamos, NM (United States); 2008 [cited 2024 Oct 31]. https://www.osti.gov/servlets/purl/960616

20 Brodersen, P.J.N. (2023) Netgraph: Publication-quality Network Visualisations in Python. *J. Open Source Softw.* **8**, 5372
21 Paysan-Lafosse, T., Blum, M., Chuguransky, S., Grego, T., Pinto, B.L., Salazar, G.A., et al. (2023) InterPro in 2022. *Nucleic Acids Res* **51**, D418–D427
22 HMMER http://hmmer.org/
23 Eddy, S.R. (2011) Accelerated Profile HMM Searches. *PLoS Comput Biol* **7**, e1002195
24 Mistry, J., Chuguransky, S., Williams, L., Qureshi, M., Salazar, G.A., Sonnhammer, E.L.L., et al. (2021) Pfam: The protein families database in 2021. *Nucleic Acids Res* **49**, D412–D419
25 Katoh, K., Rozewicki, J. and Yamada, K.D. (2019) MAFFT online service: multiple sequence alignment, interactive sequence choice and visualization. *Brief Bioinform* **20**, 1160–1166
26 Robert, X. and Gouet, P. (2014) Deciphering key features in protein structures with the new ENDscript server. *Nucleic Acids Res* **42**, W320–4
27 Jing, B., Berger, B. and Jaakkola, T. (2024) AlphaFold meets flow matching for generating protein ensembles. arXiv [q-bio.BM]. https://doi.org/10.48550/ARXIV.2402.04845
28 GitHub - lcscs12345/alphaflow: AlphaFold Meets Flow Matching for Generating Protein Ensembles. GitHub. https://github.com/lcscs12345/alphaflow
29 Lin, Z., Akin, H., Rao, R., Hie, B., Zhu, Z., Lu, W., et al. (2023) Evolutionary-scale prediction of atomic-level protein structure with a language model. *Science* **379**, 1123–1130
30 Raschka, S. (2017) BioPandas: Working with molecular structures in pandas DataFrames. *J. Open Source Softw.* **2**, 279
31 Kurtzer, G.M., cclerget, Bauer, M., Kaneshiro, I., Trudgian, D. and Godlove, D. (2021) hpcng/singularity: Singularity 3.7.3. Zenodo; 2021. https://doi.org/10.5281/ZENODO.1310023
32 Kurtzer, G.M., Sochat, V. and Bauer, M.W. (2017) Singularity: Scientific containers for mobility of compute. *PLoS One* **12**, e0177459
33 Website (https://hub.docker.com/r/lcscs12345/alphaflow
34 Meng, E.C., Goddard, T.D., Pettersen, E.F., Couch, G.S., Pearson, Z.J., Morris, J.H., et al. (2023) UCSF ChimeraX: Tools for structure building and analysis. *Protein Sci* **32**, e4792
35 McGibbon, R.T., Beauchamp, K.A., Harrigan, M.P., Klein, C., Swails, J.M., Hernández, C.X., et al. (2015) MDTraj: A Modern Open Library for the Analysis of Molecular Dynamics Trajectories. *Biophys J* **109**, 1528–1532
36 GitHub - dwhswenson/contact_map: Contact map analysis for biomolecules; based on MDTraj. GitHub. https://github.com/dwhswenson/contact_map
37 Zhang, C., Shine, M., Pyle, A.M. and Zhang, Y. (2022) US-align: universal structure alignments of proteins, nucleic acids, and macromolecular complexes. *Nat Methods* **19**, 1109–1115
38 Loizides, F. and Schmidt, B. (2016) Positioning and Power in Academic Publishing: Players, Agents and Agendas: Proceedings of the 20th International Conference on Electronic Publishing. IOS Press; 2016.
39 Jette, M.A. and Wickberg, T. (2023) Architecture of the slurm workload manager. In: Lecture Notes in Computer Science. . Cham: Springer Nature Switzerland; 2023. . p. 3–23.
